# Supplementary figures and images for: GSimp: A Gibbs sampler based left-censored missing value imputation approach for metabolomics studies
Source: PLoS Comput Biol. 2018 Jan 31;14(1):e1005973. doi: 10.1371/journal.pcbi.1005973 (PMC5809088; doi:10.1371/journal.pcbi.1005973)

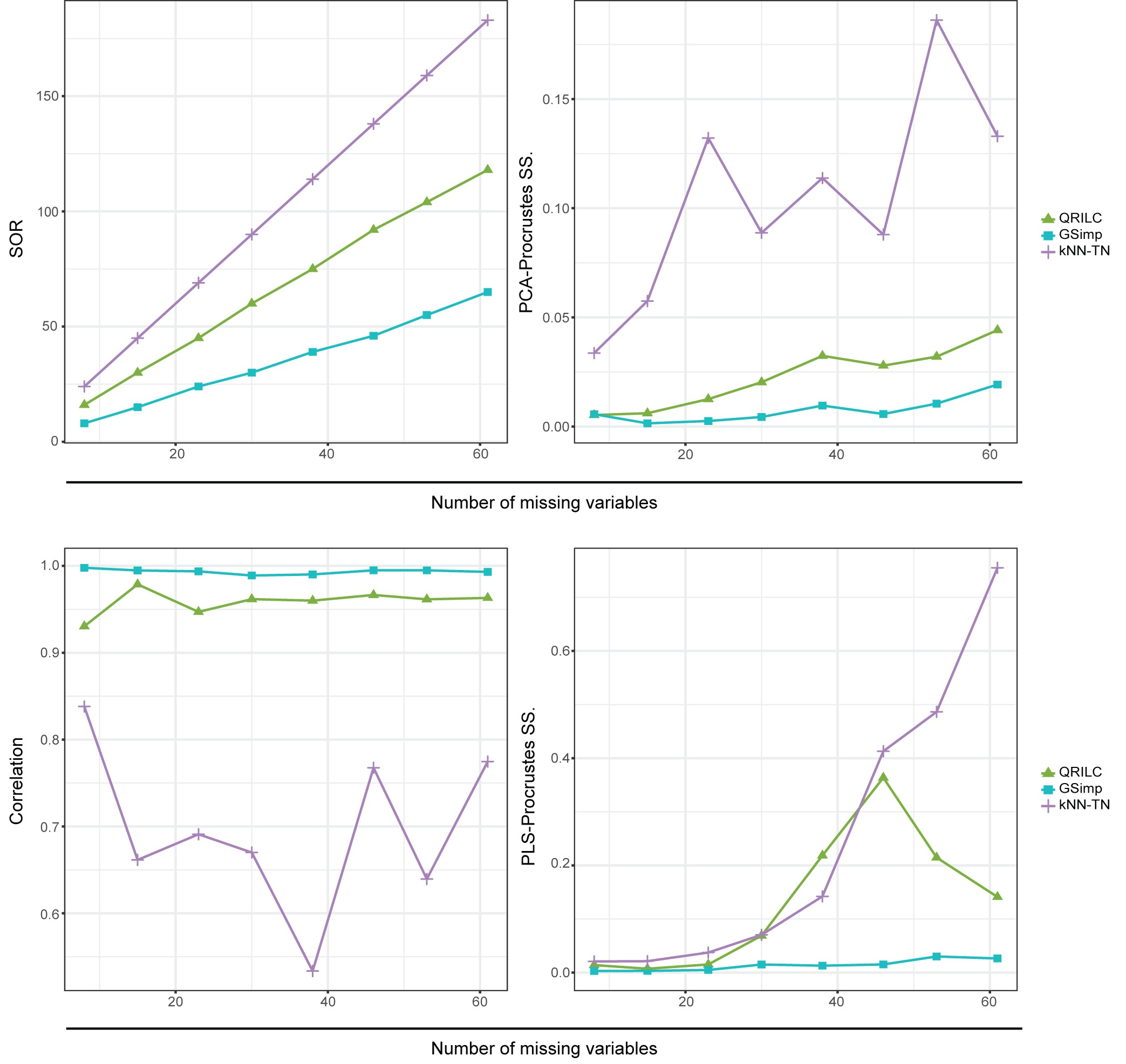

Supplement: S1 Fig — SOR (upper left), PCA-Procrustes sum of squared errors (upper right), Pearson's correlation between log-transformed p-values of student’s t-tests (lower left), and PLS-Procrustes sum of squared errors (lower right) on simulation dataset along with different numbers of missing variables based on three imputation methods: QRILC (green triangle), GSimp (blue square), and kNN-TN (purple cross). (TIF) [file pcbi.1005973.s002.tif]

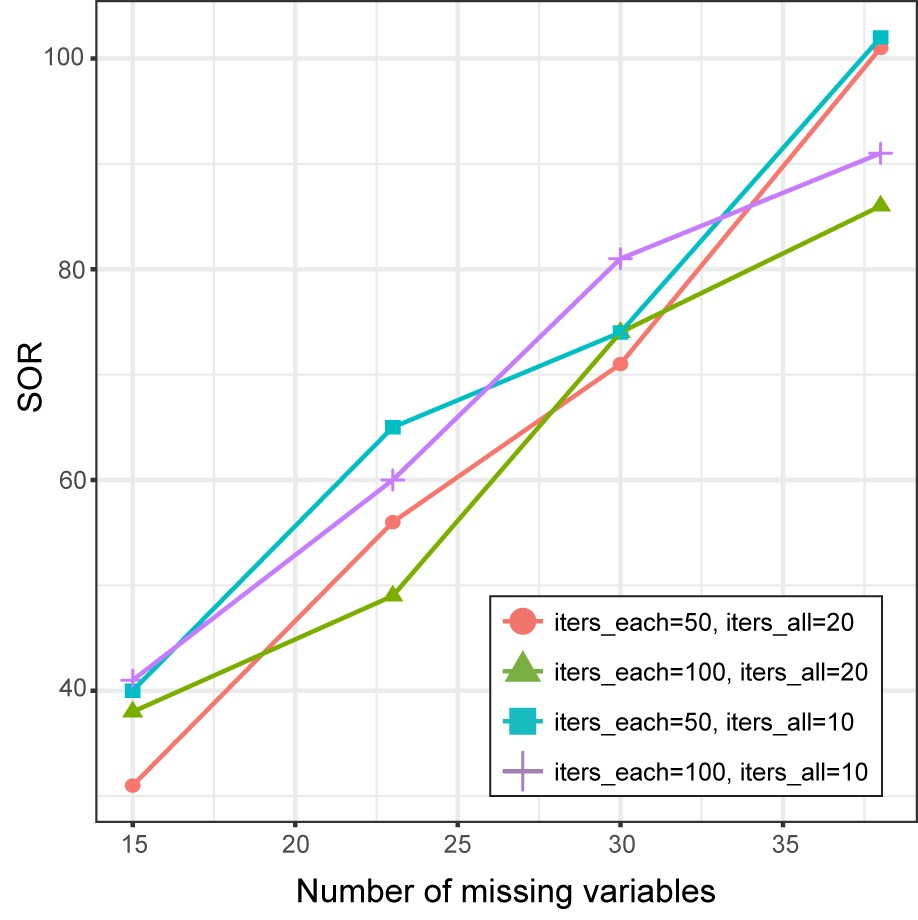

Supplement: S2 Fig — SOR on simulation dataset along with different numbers of missing variables based on four different numbers of iterations: iters_each = 50 and iters_all = 20 (red circle), iters_each = 100 and iters_all = 20 (green triangle), iters_each = 50 and iters_all = 10 (blue square), iters_each = 100 and iters_all = 10 (purple cross). (TIF) [file pcbi.1005973.s003.tif]
